# Reproducible Superinsulation Materials: Organosilica-Based Hybrid Aerogels with Flexibility Control

**DOI:** 10.3390/gels10110692

**Published:** 2024-10-25

**Authors:** Marvin Geyer, Felix Leven, Johannes Limberg, Corina Andronescu, Rainer Ostermann

**Affiliations:** 1 Technische & Makromolekulare Chemie, Westfälische Hochschule, 45665 Recklinghausen, Germany; felixleven@gmx.de (F.L.); johannes@limberg-olfen.de (J.L.); 2 Chemical Technology III, Center for Nanointegration Duisburg-Essen (CENIDE), University of Duisburg-Essen, Carl-Benz-Straße 199, 47057 Duisburg, Germany; corina.andronescu@uni-due.de

**Keywords:** hybrid aerogel, supercritical drying, bendability/flexibility control, radical polymerization, polycondensation, superinsulation material, energy storage

## Abstract

In this study, we report highly crosslinked hybrid aerogels with an organic backbone based on vinylmethyldimethoxysilane (VMDMS) with tuneable properties. For an improved and highly reproducible synthesis, a prepolymer based on 2,4,6,8-tetramethyl-2,4,6,8-tetravinylcyclotetrasiloxane (D_4_V_4_) and VMDMS as monomers was prepared and purified. Di-tert-butylperoxide (DTBP) concentrations of 1 mol% initiate the radical polymerization of the mentioned monomers to achieve high yields of polymers. After purification, the obtained viscous polyorganosilane precursor could be reproducibly crosslinked with dimethyldimethoxysilane (DMDMS) or methyltrimethoxysilane (MTMS) to form gels in benzylic alcohol (BzOH), water (H_2_O) and tetramethylammonium hydroxide (TMAOH). Whereas freeze-drying these silica-based hybrid aerogels led to high thermal conductivity (>20 mW m^−1^K^−1^) and very fragile materials, useful aerogels were obtained via solvent exchange and supercritical drying with CO_2_. The DMDMS-based aerogels exhibit enhanced compressibility (31% at 7 kPa) and low thermal conductivity (16.5 mW m^−1^K^−1^) with densities around (0.111 g cm^−3^). The use of MTMS results in aerogels with lower compressibility (21% at 7 kPa) and higher density (0.124 g cm^−3^) but excellent insulating properties (14.8 mW m^−1^K^−1^).

## 1. Introduction

Achieving carbon neutrality and decreasing energy consumption are important goals of the present world [1]. In this context, decreasing the energy consumption in residential buildings is also needed [2]. To increase energy efficiency in housing, new materials are needed that ideally show a heat conductivity below 20 mW m^−1^K^−1^ [3]. One class of high-performance insulation materials that can potentially meet these requirements are aerogels [4,5]. There are different kinds of aerogels, such as organic aerogels [6], silica aerogels [7] or titania [8]. Since aerogels were first introduced by Kistler in 1931, they represent a remarkable class of materials characterized by their ultra-low density, high porosity and exceptional thermal insulation properties [9]. These materials, composed of interconnected nanometer-scale pores within a solid matrix, have gained significant interest across various fields due to their unique combination of properties. One of the most notable features of aerogels is their extremely low density, typically ranging from 0.05 [10] to 0.30 g cm^−3^ [11], which is attributed to their high porosity, often exceeding 90% [12]. The density and structure of the aerogels have a major influence on the thermal properties of the materials. The thermal conductivity of porous materials is influenced by solid, gas and infrared (IR) components, and coupling effects can also occur [13]. In aerogels, the density is ideally adjusted in such a way that the solid component enabling the heat conduction is minimized, while at the same time maintaining a fine structure with small pore sizes [14], which results in the Knudsen [15] effect. However, high manufacturing costs [16] due to supercritical drying and adverse properties like silica aerogel’s brittleness are limiting [17] the use of aerogels to niche applications, such as space shuttle technology [18], catalysts [19] or biosensing [20,21]. Many researchers have tried to find ways to lower the cost without significantly lowering the performance; very recently, Brouwers et al. and Gurlo et al. reported using ambient pressure drying (APD) for silica aerogels from waste glass [22,23]. Hybrid aerogels present a promising path to overcome the poor mechanical performance. Leventis et al. studied the post-crosslinking of silica gels with isocyanates to produce mechanically robust aerogels with the tradeoff of higher thermal conductivity [24]. Kanamori and Nakanishi nicely reviewed the various hybridization strategies [25] and also contributed to the development of organo-substituted alkoxy silanes and organo-bridged alkoxysilanes used to create polyorganosiloxane/silsesquioxane aerogels with improved mechanical properties [26,27]. More recently, they presented a two-step approach to doubly cross-link polyvinylpolymethylsiloxane (PVMDMS) aerogels, starting with a radical polymerization of vinyldimethylmethoxysilane (VDMMS) followed by condensation with additional MTMS monomer or a radical polymerization of VMDMS followed by direct intermolecular polycondensation between the methoxy groups. The hybrid aerogels were reported to have high flexibility and low thermal conductivity, even without supercritical drying [28,29]. Following this approach, Wang et al. studied the thermal conductivity of these materials in great detail and achieved 22 mW m^−1^K^−1^ at a density of 0.177 g cm^−3^ [30]. Other groups also adapted the PVMDMS-based synthesis; Kong et al. investigated the use of methylmethacrylate (MMA) as a copolymer and found improved mechanical properties that came at the cost of their insulation properties (>30 mW m^−1^K^−1^) [31]. The same trend was also obtained by Li et al., who used PVMDMS in benzoxazine-based aerogels that are not high-performance insulation materials either (>40 mW m^−1^K^−1^) [32].

Here, we reinvestigated the synthesis of highly flexible PVMDMS-based aerogels, aiming to allow ambient pressure drying or advanced freeze-drying processes [33,34,35] of silica aerogels [36] to avoid the limitations of supercritical drying that is predominantly used for the preparation of aerogels [37,38]. Unable to reproduce the results from Kanamori et al. [28,29], we put our focus on the prepolymer synthesis, optimizing yields and purification to improve the reproducibility of the subsequent aerogel preparation [39]. Furthermore, we elucidated the importance of adding monomeric VMDMS or DMDMS [40] to obtain and enhance flexibility and adding MTMS to obtain finer structures and lower heat conductivity.

## 2. Results and Discussion

### 2.1. Radical Polymerization of PVMDMS and PV_4_D_4_VMDMS

**Figure 1:**
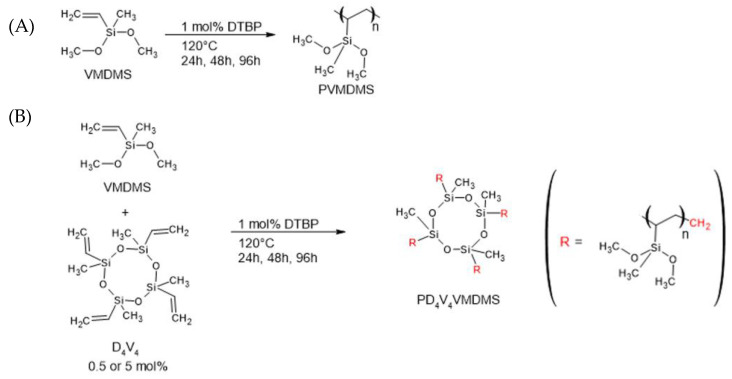
(**A**) Radical polymerization of VMDMS with DTBP as initiator. (**B**) Radical copolymerization of VMDMS and D_4_V_4_ with DTBP as initiator.

**Figure 2:**
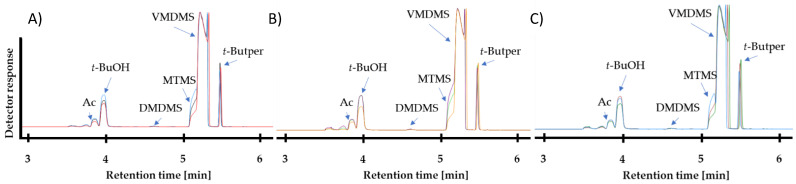
(**A**) Elugram after the radical polymerization between VMDMS, with *t*-Butper as initiator for 24 h (red), 48 h (black) or 96 h (blue). (**B**) Elugram after the radical copolymerization between VMDMS:D_4_V_4_ (ratio: 99.5:0.5), with *t*-Butper as initiator for 24 h (orange), 48 h (green) or 96 h (purple). (**C**) Elugram after the radical copolymerization between VMDMS:D_4_V_4_ (ratio: 95:5), with *t*-Butper as initiator for 24 h (dark green), 48 h (brown) or 96 h (dark blue). Abbreviations: Ac (acetone), *t*-BuOH, DMDMS, MTMS, VMDMS and t-Butper.

The radical polymerizations of VMDMS and the radical copolymerization between D_4_V_4_ and VMDMS (Figure 1) were compared for qualitative and quantitative evaluation (Table 1 and Figure 2). Here, D_4_V_4_, rich in vinyl groups, has a higher possibility to participate in radical polymerizations and thereby enhance the yields of higher molecular mass polymers. As VMDMS has a boiling point of 106 °C and, therefore, a considerable vapor pressure at the polymerization temperature of 120 °C, the vials were weighed before and after the reaction to ensure that the used GC-HS vials (Figure S1) were leakproof. A mass loss of <2% was determined over 96 h at 120 °C [41].

Additionally, the reaction products of the radical polymerization of the prepolymer were analyzed via GC–MS before purification. Here, the chromatogram shows significant impurities from the educts and the by-products of the reaction, including DMDMS at 4.6 min and MTMS at 5.1 min retention time (Figure 2A–C). By analyzing the educt, we found that MTMS and DMDMS are already present as impurities in the VMDMS monomer (only 97% purity). Additionally, *tert*-Butanol (*t*-BuOH) at 3.95 min, unreacted *tert*-Butylperoxide (*t*-Butper) at 5.5 min and VMDMS residues at 5.2 min were found. Here, *t*-BuOH is rising because more *t*-Butper had decomposed to the former with time. While the retention time strongly depends on the used GC–MS method, broad signals indicate the presence of high concentrations of VMDMS. The purification of the prepolymer in a high vacuum at 70 °C removes all volatile impurities, which is accompanied by an increase in viscosity (Table S1 and Figure S2) [42,43]. The successful removal of the impurities and by-products can be confirmed by the mass difference occurring before and after the vacuum treatment. After purification, 20–40% of the weight was lost.

The results of radical polymerization differ greatly depending on whether D_4_V_4_ was used or not. As expected, even small amounts of the higher-functional D_4_V_4_ lead to a rapid increase in viscosity due to the partial cross-linking of the VMDMS chains. Copolymers with 5 wt% D_4_V_4_ crosslink to form a gel during polymerization and cannot be redissolved in BzOH. The best results were obtained with a ratio of 99.5:0.5 (VMDMS:D_4_V_4_), which is why this ratio was used for further experiments. The ratio of 95:5 (VMDMS:D_4_V_4_) shows the highest cross-coupling degree, where the polymer does not move and is not soluble anymore.

The purified prepolymers were analyzed by ^13^C-NMR; Figure S3A shows the comparison of the PVMDMS to the VMDMS monomer. In the copolymerization (Figure S3B), a small signal of assumed vinyl groups could be found at 132 ppm after purification. As this cannot be removed even by longer purification time, it can be assumed that some C=C double bonds are still present in the finished prepolymer.

GPC measurements were carried out to further analyze the prepolymer formation. The average molar mass appears to correlate with the reaction time, but the radical polymerization is subject to strong fluctuations. Taking the error bars into account, little differentiation can be made (Figure S4). In principle, however, the measured values, like conversion and PDI, are comparable to those in the comparative literature (Table S2 vs. Figure S4). Small deviations might be attributed to different conditions or the usage of different devices. Additionally, the M_n_ of Exp. 2_2, 2_4 and 2_6 is not reported because the polymer was not dissolvable in dimethylacetamide (DMAc) and 0.01 M potassium bromide (KBr).

**Figure 3:**
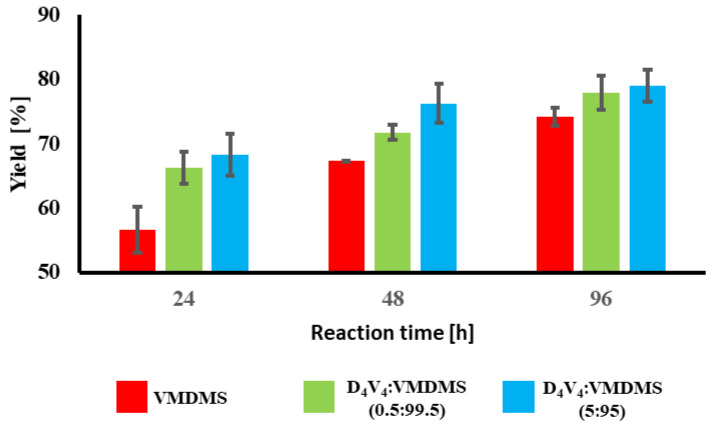
After-purification yields plotted against the reaction time of the radical polymerization (VMDMS in red) or co-radical polymerization (D_4_V_4_VMDMS: 0.5_99.5 in green and D_4_V_4_VMDMS: 0.5_99.5 in blue) for 24, 48 and 96 h. Error bars are included in each bar chart.

Tetrafunctional D_4_V_4_ polymerization leads to higher yields (Figure 3), but also induces crosslinking [39]. Comparing three different VMDMS:D_4_V_4_ ratios of 99.5:0.5, 97.5:2.5 and 95:5, we chose the first as it showed good yield already after 24 h and no adverse effects, whereas the copolymer completely crosslinks at a ratio of 95:5. While a complete analysis is beyond the scope of this work, further results from IR spectroscopy and difference scanning calorimetry (DSC) are discussed in Figures S1E1–E3 and S5.

In conclusion, a longer reaction time leads to higher yields for this radical polymerization, but yields do not significantly rise after 96 h. Nevertheless, adding and increasing D_4_V_4_ shows higher yields (0.5:99.5 of D_4_V_4_:VMDMS with 66–78%) in comparison to pure VMDMS (57–74%) as monomer. Additionally, using 2.5 mol% of D_4_V_4_ leads to viscous polymer as well. The reaction time of 48 h results in 74% ± 1% yield.

### 2.2. Polycondensation of PVMDMS

**Figure 4:**
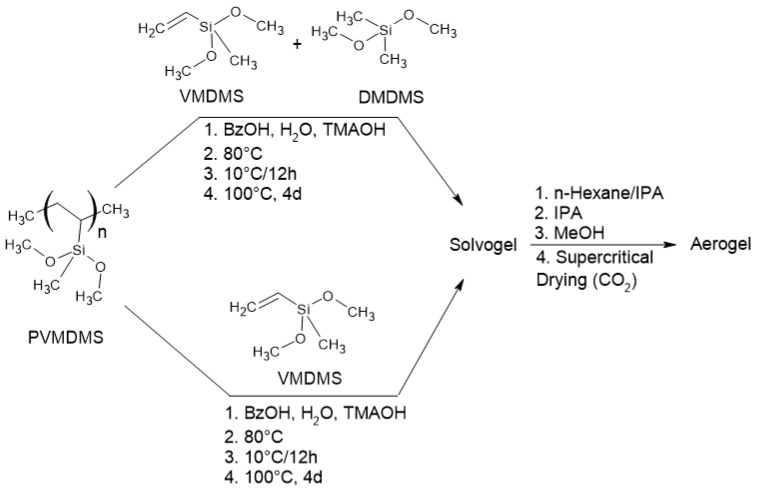
Polycondensation of PVMDMS with subsequent solvent exchange and drying protocol.

**Figure 5:**
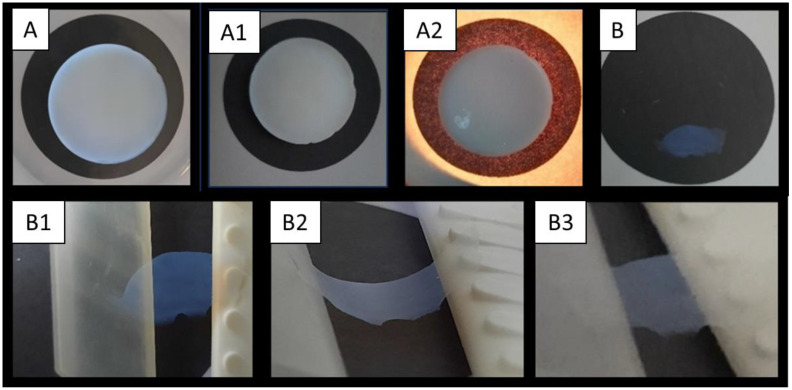
Wet solvogel in MeOH from Exp. 1 (**A**). Dried gel after supercritical drying with thickness of 4.8 mm (**A1**). Same sample illuminated to show the translucency (**A2**). Dried gel after supercritical drying with thickness of 0.1 mm (**B**). Manual bending test of the hybrid aerogel from picture B ((**B1**) = unbent, (**B2**) = curved, (**B3**) = unbent).

For the synthesis of an aerogel that is suitable for inexpensive ambient drying, flexible and mechanically robust samples should be prepared. For this purpose, Nakanishi’s and Kanamori’s approach was followed [28]. Using the purified prepolymer PVDMDS with one-part MTMS led to supercritical dried aerogels with good optical properties, but no relevant flexibility could be determined even at relatively low densities (at 0.170 g cm^−3^, the compressibility was below 5%, and testing led to the cracking of the samples). The PVMDMS mixed with one-part MTMS (6-parts BzOH/Si mol mol^−1^, 2.8-parts H_2_O/Si mol mol^−1^ and 0.03-parts TMAOH mol mol^−1^) sample gave thermal conductivity of 15.1 mW m^−1^K^−1^. The porosity measurement of this sample by BET led to a surface area of 217 m^2^ g^−1^. Adding DMDMS to increase flexibility led to more flexible (Table 2). Similar results were achieved with PVMDMS and additional non-prepolymerized VMDMS or VMDMS + DMDMS mixtures (Figure 4), but more opaque materials—most pronounced for those with DMDMS (Figure 5).

The experiments (Table 2, Exp. 1–10) were divided into two sections (dashed line). In each section, the density was varied to study the effect on compressibility. Therefore, the BzOH amount was increased, and the amounts of the other reagents were aligned to keep the concentration of water for each experiment constant. The amounts of VMDMS and DMDMS were increased with respect to the amount of lost volatile agents found in GC–MS and the obtained yields (Table 1).

To further adjust the density, the BzOH amount was varied, and the amounts of the other reagents were adjusted as well (constant concentration of water). Lowering the density leads to higher compressibility [44]. As DMDMS was found in the GC–MS analysis, it was added to the synthesis, showing increased flexibility (Figure 5B1–B3) in agreement with Hayase et al. [45]. However, the thermal conductivity increased significantly. We could reduce this adverse effect when using the D_4_V_4_-copolymer.

It can be assumed that while MTMS leads to crosslinking, the higher organic content in VMDMS and DMDMS leads to weaker interactions between the aerogel strands and thus enables flexibility [46]. Table 2 and Figure 5 show a selection of the results of different aerogel compositions in terms of density, thermal conductivity and compressibility. The tests show a strong dependence of the properties on the density. This can be adjusted primarily via the amount of solvent used in the polycondensation. Highest compression is achieved with 26% by increased monomer content of VMDMS (C1), and the thermal conductivity rose to 18.9 mW m^−1^K^−1^ with a higher solvent amount. Experiment 1 showed a very high density (0.312 g cm^−3^) and poor compressibility (8%), even if the material is still highly bendable at a low thickness (Figure 5B–B3). Low thickness results in 80% transparency, measured with 550 nm on a UV–Vis spectrometer. Sample A was measured with <1% transparency at 4.8 mm thickness. Decreasing the density by the use of higher BzOH content (see Table 2) and using co-monomers of VMDMS or DMDMS correlate with visible light transmittance in <1% at 3 mm as well.

By adjusting the amount of solvent (1.2 times the amount, e.g., Ex_1 to Ex_2), the density could be reduced and the flexibility increased. With the appropriate amounts of solvent, the desired densities between 0.100 and 0.200 g cm^−3^ were achieved.

The slow temperature increase (10 K/12 h) is particularly important with these low densities, as otherwise stress cracks will occur in the material, which can destroy the monolith [47]. During the subsequent supercritical drying via CO_2_, a shrinkage of the samples by <5% can be observed.

Depending on the density and composition, the samples show a more or less flexible behavior. Low densities and higher co-monomer contents lead to better flexibility. After compression, the monoliths showed partial recovery. In the course of the first 10 loading cycles, a total of 5–10% of the thickness is lost, after which the material remains mostly unchanged (change < 1%). A recovery to a higher volume after complete drying [48] or gentle heating [31] could not be observed. If the solvent is simply evaporated from the solvogel instead of supercritical drying, no spring-back effect is observed, and a denser xerogel is formed [48].

The thermal conductivity of the aerogels increases with the amount of co-monomer used. Most probably, the poorer insulation properties are due to larger pores and a lower Knudsen effect [15].

Further results from the IR spectroscopy and hydrophobicity are discussed in Figures S6 and S7.

### 2.3. Polycondensation of PD_4_V_4_VMDMS with MTMS or DMDMS

**Figure 6:**
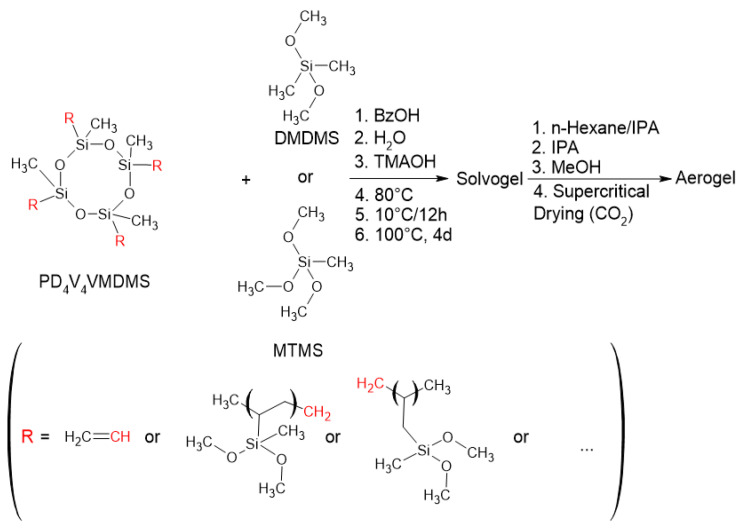
Polycondensation of PD_4_V_4_VMDMS with additional DMDMS or MTMS units.

**Figure 7:**
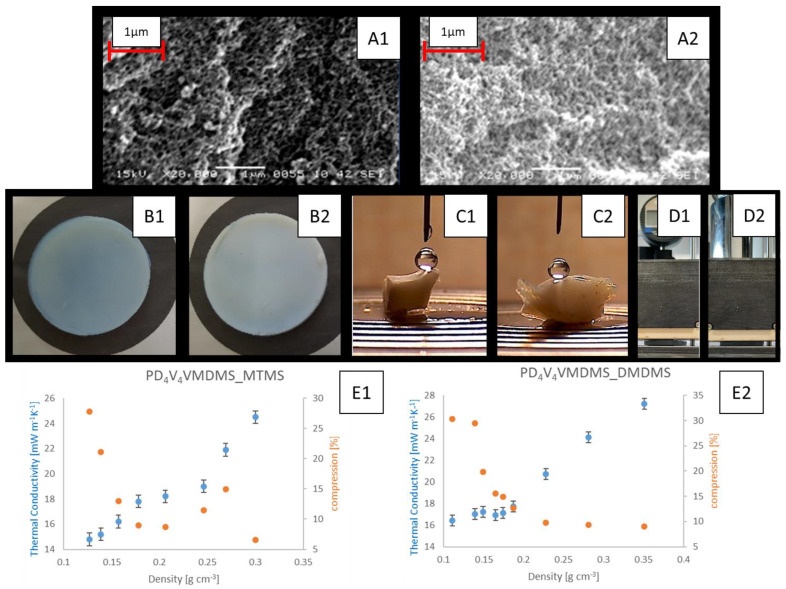
SEM images and the corresponding aerogels ((**A1**,**B1**) Exp. 5 and (**A2**,**B2**) Exp. 15) and contact angle pictures ((**C1**) Exp. 7 and (**C2**) Exp. 17) based on D_4_V_4_VMDMS backbone. On the right side, **D1** is the uncompressed aerogel and **D2** the compressed aerogel from Exp. 17. The lower panel illustrates the thermal conductivities on Y1-axis and compression on Y2-axis vs. densities for (**E1**) PD_4_V_4_VMDMS_MTMS and (**E2**) PD_4_V_4_VMDMS_DMDMS aerogels.

Based on the above findings, a polycondensation with poly-2,4,6,8-tetravinyl-2,4,6,8-tetramethylcyclotetrasiloxane-vinylmethyldimethoxysilane (PD_4_V_4_VMDMS) as a prepolymer was conducted, utilizing 1 mol/Si-unit MTMS or 0.5 mol/Si-unit DMDMS (higher DMDMS ratios resulted in decreased transparency in comparison to MTMS) as co-monomers according to Figure 6. During polycondensation, these samples are even more sensitive to shrinkage, necessitating a gradual temperature increase. On the other hand, stress cracks were not observed during polycondensation, and the resulting aerogels after supercritical drying in CO_2_ were significantly more stable compared to PVMDMS aerogels (Figure 7). The amounts of BzOH and H_2_O were adjusted to obtain the desired density (see Table S3). Herein, with respect to Table S3, increasing H_2_O as a reaction partner, adjusting BzOH as solvent and TMAOH as catalyst allows decreasing the density and keeping the concentrations of non-polymer contents constant.

For the monoliths incorporating MTMS, a very low thermal conductivity of 14.8 mW m^−1^K^−1^ at a density of 0.127 g cm^−3^ (Table S3 and Figure 7) was achieved. In comparison, the incorporation of DMDMS monomers resulted in a minimal thermal conductivity of 16.4 mW m^−1^K^−1^ at a density of 0.111 g cm^−3^. During the thermal conductivity measurements, a weight of 1.3 kg was placed on the samples for improved contact with the measurement device (error of <0.5 mW m^−1^K^−1^ compared to 1 mW m^−1^K^−1^ without the weight). This leads to compression and a higher density; however, the uncompressed density is reported.

The results also indicate that the network becomes flexible by lowering densities. In the case of PD_4_V_4_VMDMS with MTMS, a maximum compressibility of 21% was achieved; for PD_4_V_4_VMDMS with DMDMS, this was max. 30% under 1.3 kg (i.e., 7 kPa ± 1 kPa pressure, see Figure 7D1,D2). Again, there is a difference between DMDMS and MTMS—lowering the density leads to an increase in compressibility from 9 to 30% for DMDMS and 6 to 27% for MTMS. Interestingly, for MTMS aerogels, there is an unexpectedly high compression for densities around 260 g cm^−3^; between the areas of sample 2 and 6, the compression increases. Apparently, the MTMS aerogel porosity features of the material impact a higher matrix surface and introduce less volumetric space. Smaller gaps result in less compressibility.

According to the porosity data, we assume an equilibrium shift between the density and highly crosslinked microporosity phase in the aerogel lattice, which might explain the compression decrease with the use of MTMS (samples 2–6) and its subsequent increase with much lower density (samples 6 to 8). Compared to the DMDMS, less cross-linkage induced by two methoxy groups does not show this compression effect.

Similarly, hardness increases with density and the addition of MTMS—measuring shore 0 for MTMS, values of 48 ± 1 and 53 ± 1 were obtained for the densities of 160 g cm^−3^ and 275 g cm^−3^, respectively, whereas for DMDMS samples, these values were 43 ± 1 and 48 ± 1, respectively, at these densities. These values are comparable to the recently published values for PDMS (Polydimethylsiloxane that can be obtained from DMDMS), revealing a value of 44 for shore A [49].

Figure 7A1,A2 also shows the SEM images and photos of the monoliths obtained in two different experiments. Representative SEM images show a homogeneous porous structure, but the resolution is not sufficient to investigate the fine structure.

The thermal conductivities of the PD_4_V_4_VMDMS_MTMS and PD_4_V_4_VMDMS_DMDMS copolymers were plotted as a function of their density (Figure 7). It is noticeable that the insulation capacity increases with decreasing density up to a certain point. If the density is too low (PD_4_V_4_VMDMS_DMDMS < 0.150 g cm^−3^), the pore size increases, which weakens the Knudsen effect, and the radiation contribution of the heat conduction also begins to have a relevant influence on the overall thermal conductivity [15].

In addition, Figure 7 shows images obtained during the contact angle measurements for Table S3, Experiments 7 and 17.

All samples were hydrophobic. On the as-synthesized aerogel surface, the contact angles, measured using the sessile drop method with waterdrops, were consistently at 88° ± 8° for all the samples with MTMS as a co-monomer and at 82° ± 5° for DMDMS on the relatively smooth surface. In contrast, by cutting and measuring the contact angles on an inner surface, values up to 140° for MTMS and 125° for DMDMS-based aerogels were observed, indicating a Cassie–Baxter regime for the porous structure in the bulk [50].

Using the Brunauer Emmett Teller (BET) analysis reveals an increase in surface area when the densities are lowered, i.e., with increasing solvent amounts. For example, the DMDMS surface area increases from 465 m^2^ g^−1^ to 702 m^2^ g^−1^ (samples 12 and 17) and from 901 m^2^ g^−1^ to 1307 m^2^ g^−1^ (samples 2 and 7) when density is reduced from 275 g cm^−3^ to 160 g cm^−3^.

Better insulation properties are also due to smaller pores, i.e., finer structures [15]. This correlates with an increase in transmittance, as measured with a 3 mm-thick sample at constant 550 nm on a UV–visible photometer. For example, at a density of 160 g cm^−3^, the transmittance increases from 31% to 78% when using MTMS instead of DMDMS (see Table S3, samples 12 and 2). At a higher density of 275 g cm^−3^, the transmittance increases from <1% to 12% when using MTMS instead of DMDMS (see Table S3, samples 7 and 17).

The thermal stability of the aerogels was determined via TGA (thermogravimetry analysis), taking the onset of decomposition as the temperature when a mass loss of the organic portion occurs. It was found that the MTMS copolymers (357–380 °C) have a slightly higher thermal stability than those with DMDMS (328–361 °C). A lower organic content favors decomposition at higher temperatures. In any case, the thermal stability is far above the application temperature of most aerogel materials (cryogenic to ambient) and is therefore of no particular interest. Exemplary diagrams can be found in Figure S8. The results from IR spectroscopy are shown in Figure S9.

The results from the freeze-drying of D_4_V_4_ aerogels are shortly presented in the Supporting Information, Figures S10 and S11; they mostly led to cracked or fragmented aerogels, rather than monoliths. Our preliminary conclusion that the hybrid materials do not tolerate the freezing process will be further discussed in a separate publication of successfully freeze-drying other aerogels as monoliths.

## 3. Conclusions

We studied the synthesis of doubly cross-linked polyvinylpolymethylsiloxanes with a focus on the qualitative and quantitative analysis of the products from the radical (co)polymerization of VMDMS, introducing D_4_V_4_ as a useful co-monomer that increases yield and molecular weight and allows the addition of DMDMS to increase flexibility almost without increasing thermal conductivity.

As VMDMS does not polymerize well, unreacted monomers and by-products that were found in significantly varying amounts could be removed by degassing in high vacuum leading to reproducible prepolymer synthesis and properties. Using either MTMS or DMDMS (or even controlled amounts of monomer VMDMS) in the subsequent condensation, hybrid aerogels with either good insulating properties (14.8 mW m^−1^K^−1^, 0.124 g cm^−3^, 21% compressible at 7 kPa) or good compressibility (16.5 mW m^−1^K^−1^, 0.111 g cm^−3^, 31% compressible at 7 kPa) were obtained in a reproducible manner. Without purification, the varying amounts of prepolymer, residual monomer and other components resulted in aerogels with varying properties and quality.

## 4. Materials and Methods

**Chemicals:**

Chemicals were used as received. Vinylmethyldimethoxysilane (97%) and 2,4,6,8-Tetravinyl-2,4,6,8-tetramethylcyclotetrasiloxane (97%) were purchased from Alfa Aesar, Germany. Dimethyldimethoxysilane was obtained from Dowcorning, Germany. Benzylic alcohol, di-tert-butylperoxide and tetramethylammonium hydroxide (25 wt% in H_2_O) were obtained from Sigma-Aldrich, Germany. Nitrogen and liquid carbon dioxide were purchased from Linde AG Germany. Methanol and n-hexane were purchased from VWR Chemicals, Germany. Technical isopropanol was from Evonik Industries AG, Germany.

**Characterization:**

The molecular weight number (Mn) of the polymers and polydispersity index (PDI) were obtained using gel permeation chromatography (GPC) with dimethylacetamide (DMAc) and 0.01 M potassium bromide (KBr); PMMA was used as a calibration standard. A PSS GRAM analytical linear (8 × 300 mm) 10 µm, polyester copolymer column and a Knauer manual injector were used. ^13^C-NMR (nuclear magnetic resonance) was performed with an Ascend 400 MHz spectrometer using CDCl_3_ as solvent. ^1^H-NMR (nuclear magnetic resonance) was performed with a Bruker 300 MHz spectrometer using CDCl_3_ as solvent. The bulk density of the aerogels was determined with an XP/XS balance. FT-IR spectra were collected using a Thermo NICOLET 4700 spectrometer from Thermo, Madison WI, USA. The gas chromatography mass spectrometer (GC–MS) used an Agilent 6890N GC (gas chromatography) coupled with a 5973 MS (mass spectrometer) network and a Multi Purpose Sampler (MPS-2) from Gerstel. Contact angle measurements were performed using a modified Krüss goniometer with a USB microscope, equipped with a 100 µL microsyringe from VWR. Rheology was studied with an Anton Paar modular compact rheometer MCR302e with external nitrogen flow source. Samples for porosimetry preparation were degassed for one day. Surface area was calculated by the Brunauer Emmett Teller Method (BET), whereas the sample was measured by Anton Paar Nova 800 BET device. Here, the measurement is performed using degassing method at 77 K. Photometry is performed on Agilent Cary 60 UV-Vis spectrophotometer at 550 nm. Shore hardness was determined with shore 0 Durometer.

Thermogravimetric (TG) measurements were taken with a TG209 F1 Libra device and Difference Scanning Calorimetry (DSC) measurement using a DSC214 polyma, both from Netzsch. The heat conductivity was measured using a Thermtest HFM25 with XPS (extruded polystyrene) as calibration standard. To perform a powder thermal conductivity, the freeze-dried monolith is wrapped in plastic bottle and grinded mechanically before scanning. The Thermtest HFM25 has an integrated diameter to determine the compression. Gas chromatography-head space (GC-HS) vials from avantor delivered by VWR were used as reactors. Here, a headspace vial cap equipped with an 18 mm magnetic screw thread cap with PTFE/Silicone 100/PAK or headspace vial caps with 18 mm magnetic screw thread cap with PTFE/Red Chlorobutyl, 100/PAK, with PTFE/Red Chlorobutyl, 100/PAK caps (Figure S1) were used. The polycondensation reactions were performed in tri-clamp container with teflon sealing.

**Radical polymerization of VMDMS:** A 20 mL GC headspace vial was flushed with nitrogen (N_2_). Subsequently, VMDMS and 1 mol % DTBP initiator were transferred to the vial and harshly mixed for 1 min by vortex. After closing the vial tightly, the solution was heated up to 120 °C for 24 h, 48 h or 96 h (see Table 1). The obtained VMDMS prepolymer (PVMDMS) was purified from the viscous solution in a 1 × 10^−2^ mbar high vacuum at 70 °C for 3 days.

**Radical copolymerization of D_4_V_4_ and VMDMS:** VMDMS and D_4_V_4_ (for ratios see Table 1) were added to a nitrogen-flushed 20 mL GC headspace vial, mixed with 1 mol % DTBP as initiator, sealed and homogenized in a vortex mixer. The solution was heated to 120 °C for 24 h, 48 h or 96 h. The resulting viscous prepolymer was purified under high vacuum at 1 × 10^−2^ mbar and 70 °C for 4 days to obtain Poly(2,4,6,8-tetravinyl-2,4,6,8-tetramethylcyclotetrasiloxane-co-vinylmethyldimethoxysilane) (PDV_4_D_4_VMDMS).

**Polycondensation of PVMDMS:** The polycondensation reactions were carried out with the viscous PVMDMS. In preparation, wrapping film was spread out on the bottom of an autoclave container (Figure S12) so that the resulting solvogel could be conveniently removed later. For the reaction, PVMDMS, DMDMS, BzOH, H_2_O and TMAOH (see Table 2) were mixed, placed in the autoclave and sealed. Gel formation and aging took place at 60 °C over a period of 12 h. We found that slow heating and slowly induced gelation prevented the formation of cracks. We further increased the temperature by 10 °C every 12 h until 100 °C was reached. The solvogel was aged for another 4 days. The autoclave was then slowly cooled and opened, and the solvent was exchanged with IPA four times at 60 °C and twice with MeOH. Finally, the solvogel was transformed into an aerogel by supercritical drying via CO_2_. The procedure for the supercritical drying is illustrated in Figures S13 and S14.

**Polycondensation of PD_4_V_4_VMDMS with DMDMS or MTMS:** The polycondensation reactions were performed analogously to polycondensation of PVMDMS. Here, PD_4_V_4_VMDMS was mixed, according to the reaction in Figure 6, with different amounts of MTMS or DMDMS (Table S3). The polycondensation of PDV_4_D_4_VMDMS with MTMS leads to the hybrid material PD_4_V_4_VMDMS_MTMS and PD_4_V_4_VMDMS with DMDMS to the hybrid material PD_4_V_4_VMDMS_DMDMS.

## Figures and Tables

**Table 1:** Radical polymerization experiments of 1 mL VMDMS and 1 mol % DTBP at 120 °C (1_1 to 1_3). Radical copolymerization experiments of VMDMS:D_4_V_4_ and 1 mol % DTBP at 120 °C (2_1 to 2_6).

Exp.	VMDMS [mL]	D_4_V_4_ [mL]	VMDMS:D_4_V_4_ [mol:mol]	Time [h]	M_n_ [g/mol]	PDI	Yield [%]
1_1	1	-	-	24	2761	1.7	57
1_2	1	-	-	48	2807	1.7	67
1_3	1	-	-	96	3054	1.7	74
2_1	1	0.012	99.5:0.5	24	3122	2.1	66
2_2	1	0.122	95:5	24	- *	-	68
2_3	1	0.012	99.5:0.5	48	3212	2.2	72
2_4	1	0.122	95:5	48	- *	-	76
2_5	1	0.012	99.5:0.5	96	3325	1.9	78
2_6	1	0.122	95:5	96	- *	-	79

* Polymer only partially soluble.

**Table 2:** PVMDMS (0.9 g) with the applied amounts of reactants in relation to the silica content and its resulting thermal conductivity, density and compressibility (Abbreviations: compressibility—compres., thermal conductivity—κ and Silicon—Si).

Exp.	VMDMS: PVMDMS [mol:mol]	DMDMS: PVMDMS [mol:mol]	BzOH/Si [mol mol^−1^]	H_2_O/Si [mol mol^−1^]	TMAOH/Si [mol mol^−1^]	κ [mW m^−1^K^−1^]	Density [g cm^−3^]	Compres. [%]
1	0.3	0.1	4.3	2.0	0.03	25.5	0.312	8
2	0.3	0.1	5.2	2.4	0.03	22.7	0.247	16
3	0.3	-	6.0	2.8	0.03	19.8	0.174	12
4	0.3	-	7.7	3.6	0.04	18.9	0.138	26
5	0.2	-	6.9	3.2	0.04	17.3	0.145	18
6	0.2	-	7.7	3.6	0.04	18.7	0.120	22
7	0.1	-	6.9	3.2	0.04	18.3	0.165	12
8	0.1	-	8.6	4.0	0.05	17.1	0.127	20
9	-	-	7.7	3.6	0.04	17.2	0.171	17
10	-	-	9.5	4.4	0.05	17.0	0.129	19

## Data Availability

All data and materials are available on request from the corresponding author. The data are not publicly available due to ongoing researches using a part of the data.

## Supplementary Materials

The supporting information can be downloaded at https://www.mdpi.com/article/10.3390/gels10110692/s1. DSC showed no glass transition temperature or occurring side reaction during heating of the polymer from 20 C until 280 °C [28]. No OH-group was observed, which means that no observable *t*-BuOH was measured at all [51]. The reason behind the slower kinetic reaction is threefold: slow decomposition of DTBP [52], steric hindrance and the inherently uncontrolled nature of radical polymerization [53]. Hereby, phase change from solid to gas does not lead to capillary forces as well and the aerogel should be unharmed after drying [36]. Performing a freeze drying in t-BuOH at other flexible organosilica aerogels is not impossible and the details will be in our next publication. There, we compare another, supercritical dried flexible aerogel from Kanamori et. al. from their recent publication (50% compression.) [54] and compare it with our advanced freeze-drying method.
