# Supplementary material for: Reproducible Superinsulation Materials: Organosilica-Based Hybrid Aerogels with Flexibility Control
Source: Gels. 2024 Oct 25;10(11):692. doi: 10.3390/gels10110692 (PMC11593423; doi:10.3390/gels10110692)
Supplement: Supplementary file 1 [file gels-10-00692-s001.zip › gels-3252925-supplementary.pdf]

Supporting Information

Reproducible superinsulation materials: organosilica-based hybrid aerogels with flexibility control

SI: Radical Polymerisation

First, we used an autoclave container with Teflon inlay as reactor. This reactor unit was not as tight as the GC vessel and >10 % of the initial content was lost after 48 h. We assume that mostly VMDMS and some by-products evaporate during the reaction time. Therefore, GC-HS vials were used for further reactions (Figure S1). Important for the success of the reaction in the GC-HS vials are the caps. By using different caps during the radical reaction in a GC-HS vial, we observed that the PTFE/Silicone caps were not as tight as the PTFE/Red Chlorobutyl ones (< 2 % weight loss after 48 h)

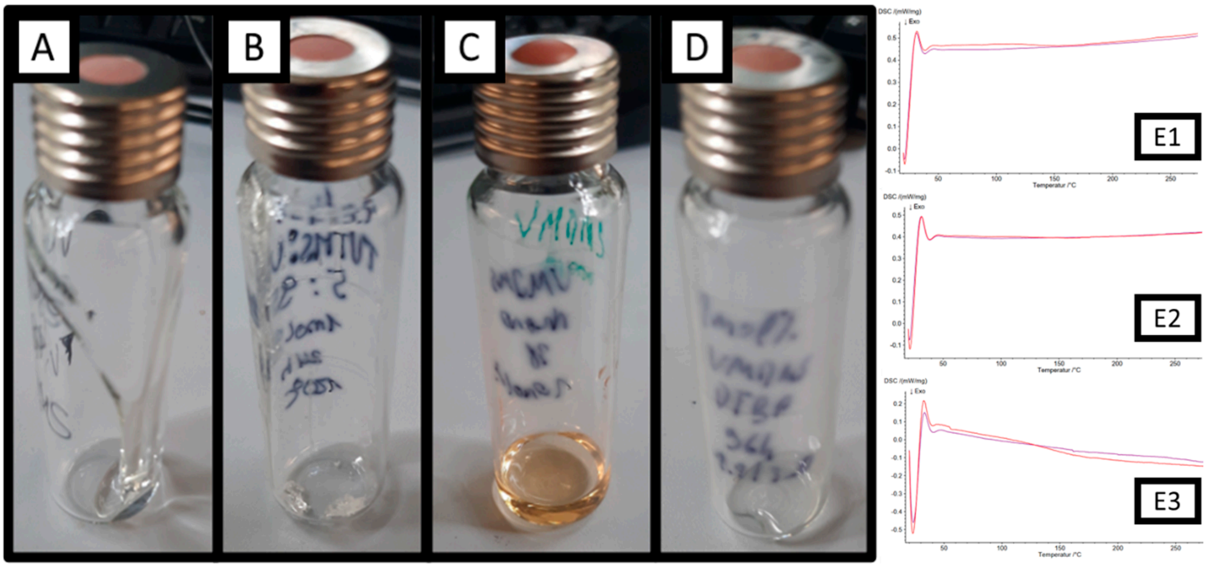

Figure S1: GC-HS vessel used for radical polymerizations. Purified polymers after the performed radical polymerization. (A) PD_4V_4VMDMS (0.5:99.5) with 1 mol% DTBP, (B) PD_4V_4VMDMS (5:95) with 1 mol% DTBP, (C) PVMDMS synthesized with 10 mol% DTBP and (D) PVMDMS with 1 mol% DTBP. DSC of the polymers from table 1 shows data for exp 1.2 (E1) and exp 2.3 (E2) and exp 2.4 (E3).

Interestingly, it became apparent that the initially colourless liquid was getting coloured when high amounts of initiator was used (Figure S1, A-D). High initiator concentration resulted in the unexpected formation of a yellowish, viscous polymer (Figure S1, C). The yellow polymer was obtained with M_n = of 2170 g/mol and a PDI of 4.93 after 48 h. The viscosities η (Table S1) of the polymers were rising with higher D_4V_4 amounts and reaction times.

Table S1: Rheometer results of the pre-polymer for the hybride Aerogel synthesis (impure pre-polymer vs purified pre-polymer).

Exp.	Reaction time [h]	VMDMS:D_4V_4 [mol:mol]	η as synthesized [mPa s]	η after purification [mPa s]
1_1	24	-	9.30	16770
1_2	48	-	16,41	21560
1_3	96	-	21.92	12960
2_1	24	99.5:0.5	26.59	21560
2_2	24	95:5	5256	-*
2_3	48	99.5:0.5	13.55	19330
2_4	48	95:5	-*	-*
2_5	96	99.5:0.5	98.78	21520
2_6	96	95:5	-*	-*

*No measurement possible.

The as-synthesized polymers contain a significant amount of monomer and impurities and therefore show low viscosities (1-100 mPa s). The copolymers with 5%. After purification the viscosity rises especially with the copolymer, proving the effectiveness of the procedure. An example for rheology measurement for purified- and impure pre-polymer is provided in Figure S2 for exp. 1_2 after 48 h.

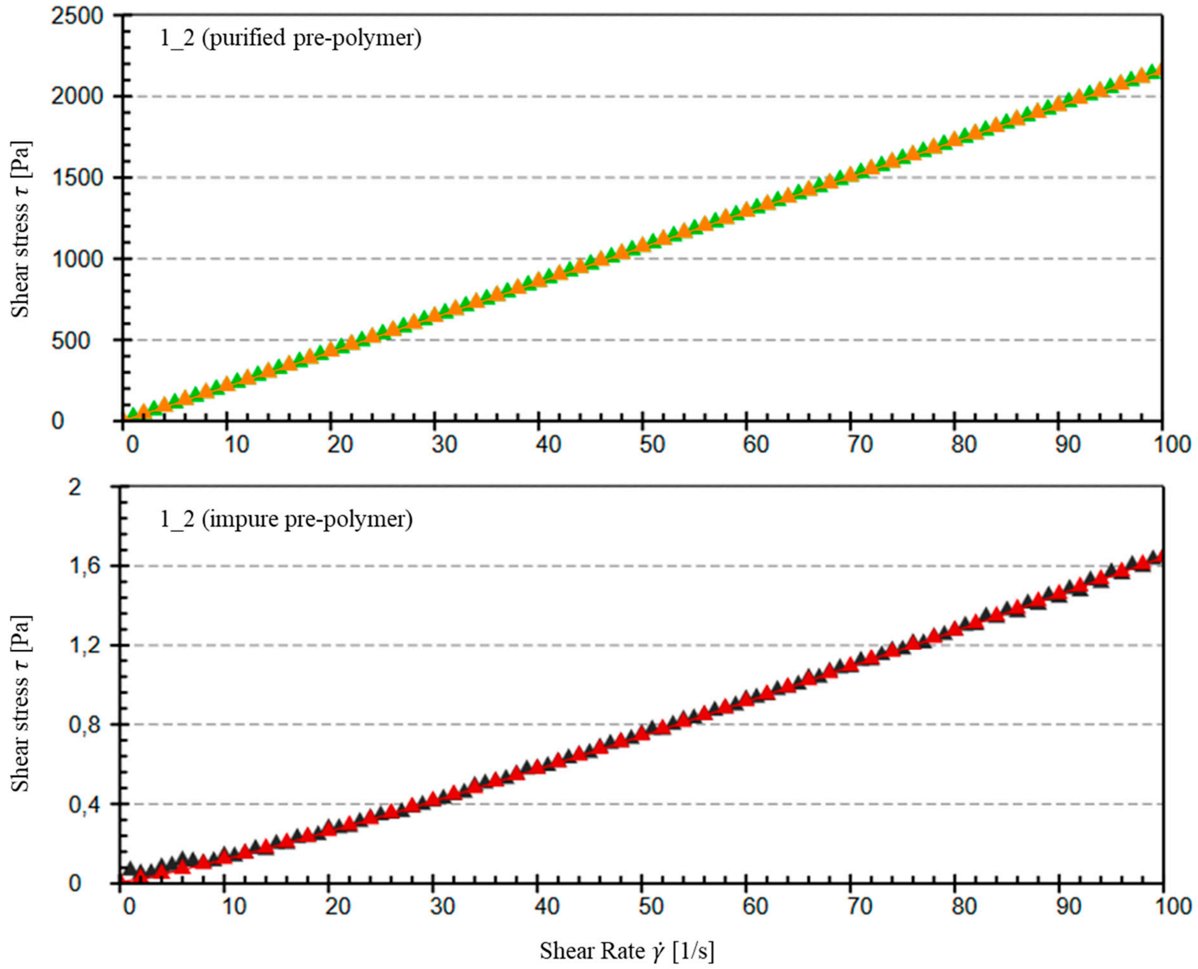

Figure S2: Example rheology measurement of the impure and purified prepolymers from Table S1 for Exp. 1_2. Shear stress is plotted against shear rate.

The viscosity plot (green) is the measurement, fitted by a regression curve (orange) after Herschel-Bulkley. The viscosity is calculated at 100 s^{-1} (see results Table S1). As can been seen from Figure S2 the plot for the purified pre-polymer is almost perfectly linear, whereas the impure pre-polymer shows more deviations (measurement black, regression red).

The more initiator the lower the polymer chain length (as theory predicts), but in contrast to the literature (table 1, Exp 1_1, 1_2 and 1_3 with Table S2). Additionally, DSC showed no glass transition temperature or occurring side reaction during heating of the polymer from 20 °C until 280 °C [28].

Table S2: Results of the radical polymerization of VMDMS with different polymerization time and DTBP concentrations from Literature [28].

DTBP [mol%]	Polymerization time [h]	Mw$^{a)}$	Degree of polymerization	Mw/Mn$^{b)}$	Conversion [%]
1	24	5358	40.5	1.86	91
1	48	5356	40.5	1.86	95
1	72	6038	45.7	2.09	99
5	48	8998	68.0	2.57	99

Weight-average molecular weight $^{a)}$. Number average molecular weight $^{b)}$.

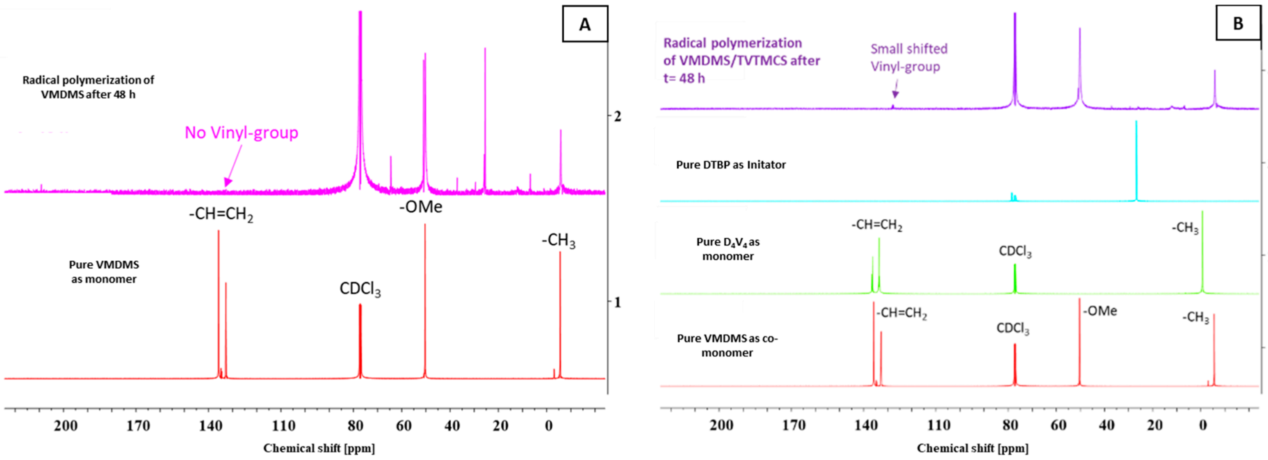

Figure S3: (A) The ^{13}C-NMR of the reactant VMDMS vs the purified viscous oil after 48 h, with disappeared double bond of the vinyl-group. (B) The ^{13}C-NMR of D_4V_4VMDMS after purification the radical copolymerization compared with the educts after 48 h, shows low concentration of not functionalized or unreacted vinyl-groups left.

Being better suited than simple ^{1}H-NMR, we chose ^{13}C NMR (Figure S3, A and B) to show successful reaction/polymerization on the vinyl group that disappears at 138 ppm after polymerisation and subsequent purification. The ^{1}H-NMR characterized the PVMDMS (300 MHz, $CDCl_3$) σ 3.48 (s, 6H), 1.52 (s, 2H), 0.70 (s, 1H), 0.10 (s, 3H). The ^{1}H-NMR for PD_4V_4VMDMS is more difficult to interpret due to irregular polymerization on 4 different vinyl positions. The D_4V_4 concentrations are to too low (especially at 0.5%) for accurate characterization and integration.

When D_4V_4 was introduced with 5 mol% to the VMDMS monomer, a more rapid polymerization occurred within the first 24 hours. This can be attributed to the increased amount of vinyl-groups, which is significantly higher compared to 0.5 mol% D_4V_4 or pure VMDMS in radical polymerization. After 48 hours, both 5 mol% D_4V_4 and 0.5 mol% VMDMS approached approximately 70 % yields and these yields remained relatively constant until the 96-hour mark. The reason behind the slower kinetic reaction is threefold: slow decomposition of DTBP [52], steric hindrance and the inherently uncontrolled nature of radical polymerization [53]. These factors decrease the interactions between vinyl groups in the cyclic siloxane functional groups, making the process more challenging. During the initial 24 to 48 hours, co-radical polymerizations quickly achieved similar yields and exhibited minimal changes thereafter. Notably, the graph illustrates lower yields for the radical polymerization of VMDMS only. Given the lack of significant changes beyond this point, we speculate termination occurring after a polymerization degree of approximately 21 till 23 repeating units, with increasing reaction time calculated over M_n-values (Figure S4). Unfortunately, the specific termination group remains unidentified. Our attempts to identify an end group through IR spectroscopy (Figure S5) or NMR proved inconclusive.

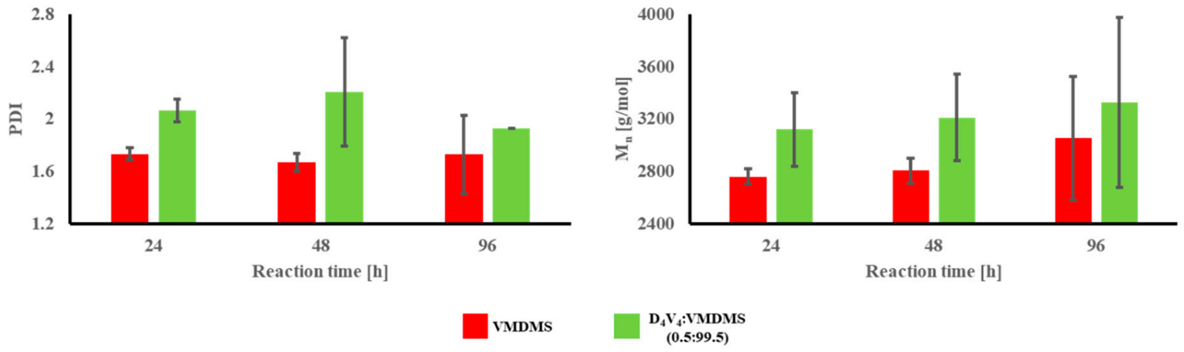

Figure S4: Molecular mass (Mn) and Polydispersity's index (PDI) plotted against the reaction time of the radical polymerization (VMDMS in red) or co-radical polymerization (D_4V_4VMDMS: 0.5_99.5 in green) for 24-,48-and 96 hours. Error bars are included in each bar chart.

In ATR-FTIR (Figure S5) absorption bands at 2935 to 2833 cm^{-1} are associated to stretching C-H bond. Assuming, 1456 to 1256 cm^{-1} to the deformation stretching of C-H bond. Bands in range of 799 and 767 cm^{-1} are proposed to be CH_3 rocking and asymmetric Si-C bonds. No OH-group was observed, which means that no observable *t*-BuOH was measured at all [51]. DTBP concentrations might be too low to be detected by IR, since in GCMS analysis it was still present.

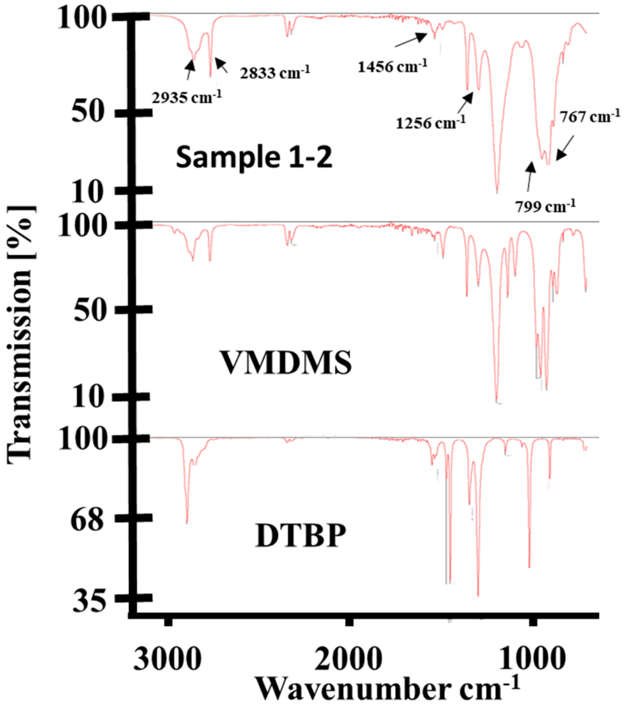

Figure S5: ATR-FTIR spectra of the radical polymerization of VMDMS for 48h at 120 °C compared with educt from table 2, exp. 1-2.

The aerogels were qualitative analysed by IR spectroscopy (Figure S6). As evidence of unreacted vinyl groups, IR spectroscopy was conducted. FTIR spectra bands for PVMDMS_VMDMS_DMDMS aerogel (Table 2, Exp. 1) showed bands comparable with those from Figure S5. A C=C stretching bond at 1560 cm^{-1}, may indicate vinyl-groups in the polymer or monomer residues. These vinyl-groups were not found in the PVMDMS spectra.

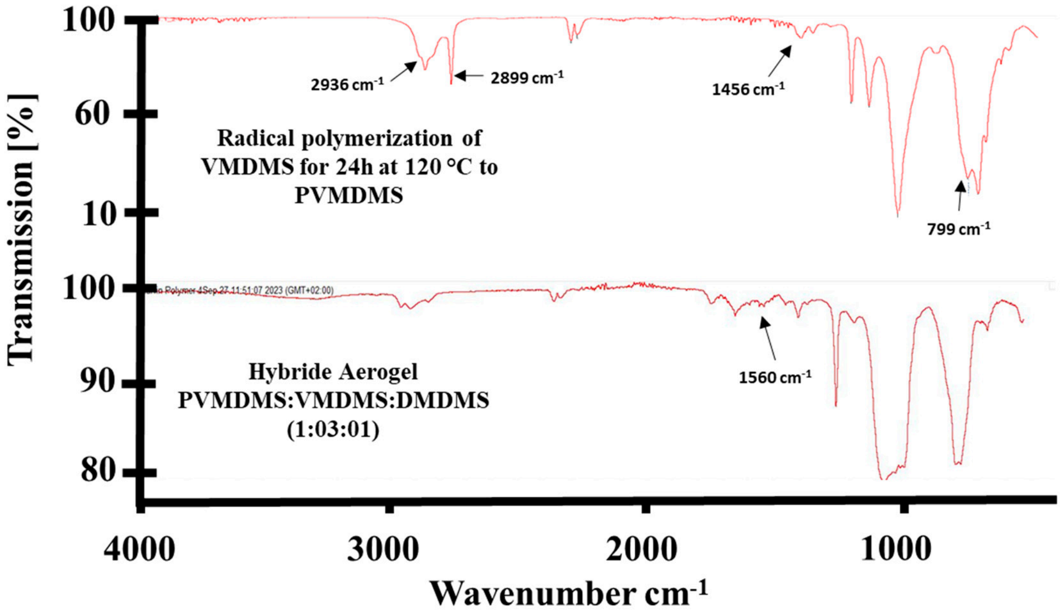

Figure S6: FTIR spectra of pure PVMDMS as pre-polymer compared with PVMDMS_VMDMS_DMDMS aerogel from paper Table 1 Exp 1.

Hydrophobicity was analysed from sessile water drops in a contact angle measurement setup (Figure S7). This method shows with increasing addition of hydrophobic monomers, i.e. VMDMS or VMDMS+DMDMS, the hydrophobicity rises. Nevertheless, the hydrophobicity was not the focus in this study.

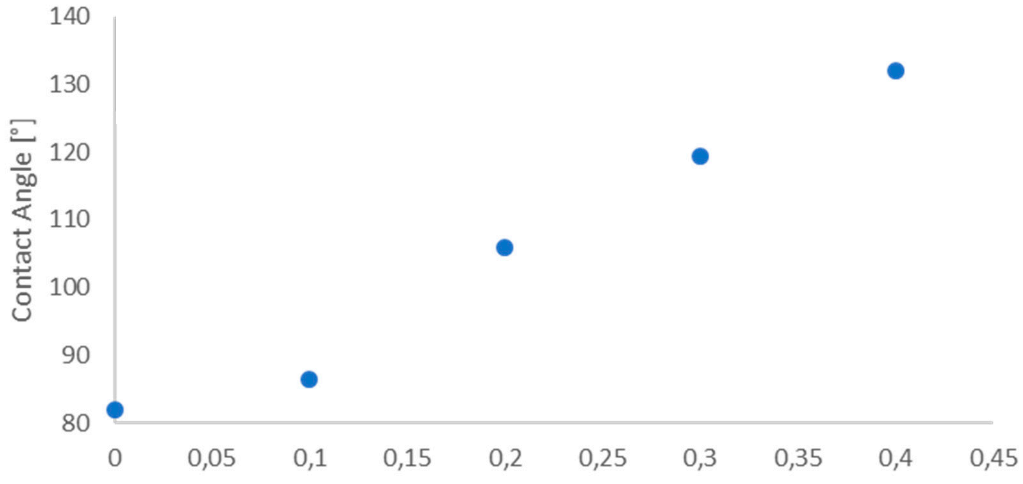

Figure S7: Results of the contact angle measurements during increasing VMDMS/PVMDMS (0, 0.1, 0.2 and 0.3) or VMDMS+DMDMS/PVMDMS (0.3+0.1) on x-axis in paper Table 2.

SI: Polycondensation of PD_4V_4VMDMS with MTMS or DMDMS

The polycondensation reactions of PD_4V_4VMDMS with MTMS or DMDMS were performed according to table S3. The reaction conditions are the same according to the Polycondensation of PVMDMS.

Table S3: Parameter for polycondensation of D_4V_4VMDMS.

Exp. PD_4V_4 VMDMS	PVMDMS/ MTMS [mol mol^{-1}]	PVMDMS/ DMDMS [mol mol^{-1}]	BzOH/Si [mol mol^{-1}]	H_2O/Si [mol mol^{-1}]	TMAOH/Si [mol mol^{-1}]	Density [g cm^{-3}]	κ [mW m^{-1}K^{-1}]
1	1.0	-	12.6	8.0	0.08	0.300	24.5
2	1.0	-	15.0	10.0	0.11	0.269	21.9
3	1.0	-	17.6	11.2	0.11	0.246	19.0
4	1.0	-	20.2	12.8	0.13	0.206	18.2
5	1.0	-	22.7	14.4	0.13	0.178	17.8
6	1.0	-	25.2	16.0	0.14	0.157	16.2
7	1.0	-	27.7	17.6	0.14	0.139	15.2
8	1.0	-	32.8	20.8	0.15	0.127	14.8
9	1.0	-	35.3	22.4	0.15	0.113	15.5
10	1.0	-	25.2	8.0	0.14	0,180	17.7
11	-	0.5	12.6	8.0	0.11	0.351	27.2
12	-	0.5	15.0	10.0	0.11	0.281	24.1
13	-	0.5	17.6	11.2	0.11	0.228	20.7
14	-	0.5	20.2	12.8	0.13	0.187	17.7
15	-	0.5	22.7	14.4	0.13	0.174	17.1
16	-	0.5	25.2	16.0	0.14	0.165	16.9
17	-	0.5	27.7	17.6	0.14	0.150	17.2
18		0.5	32.8	20.8	0.15	0.139	17.0
19	-	0.5	35.3	22.4	0.15	0.111	16.4
20	-	0.5	25.2	8.0	0.14	0,180	17.6

When measuring the thermal conductivity, the samples are loaded with 1.3 kg (7 kPa ± 1 KPa) to ensure good thermal contact with the measuring plates. This leads to a partial deviation of the measured values, as aerogels with higher flexibility are compressed more and thus have a higher density (but also smaller pores) during the measurement. Therefore, we measured both, low contact pressure (0.3 Kg) and with applied pressure of 1.3 Kg (7 kPa ± 1 KPa). With low pressure, the thermal conductivity of the aerogels is up to 1 mW m^{-1}K^{-1} higher. Also, studying the water contents assumes less H_2O as a reagent, less crosslinking, and therefore, higher densities and thermal conductivities (Table S3, Experiments 10 and 19). The lower densities obtained in the case of MTMS-based aerogels resulted in increasing thermal conductivity (Table S3, Experiment 9), whereas similar DMDMS-based aerogels were not stable in higher dilutions and couldn´t be measured anymore.

Figure S8 illustrates the TG results of the aerogels. The decomposition temperature was defined as the temperature when mass loss of organic content occurs.

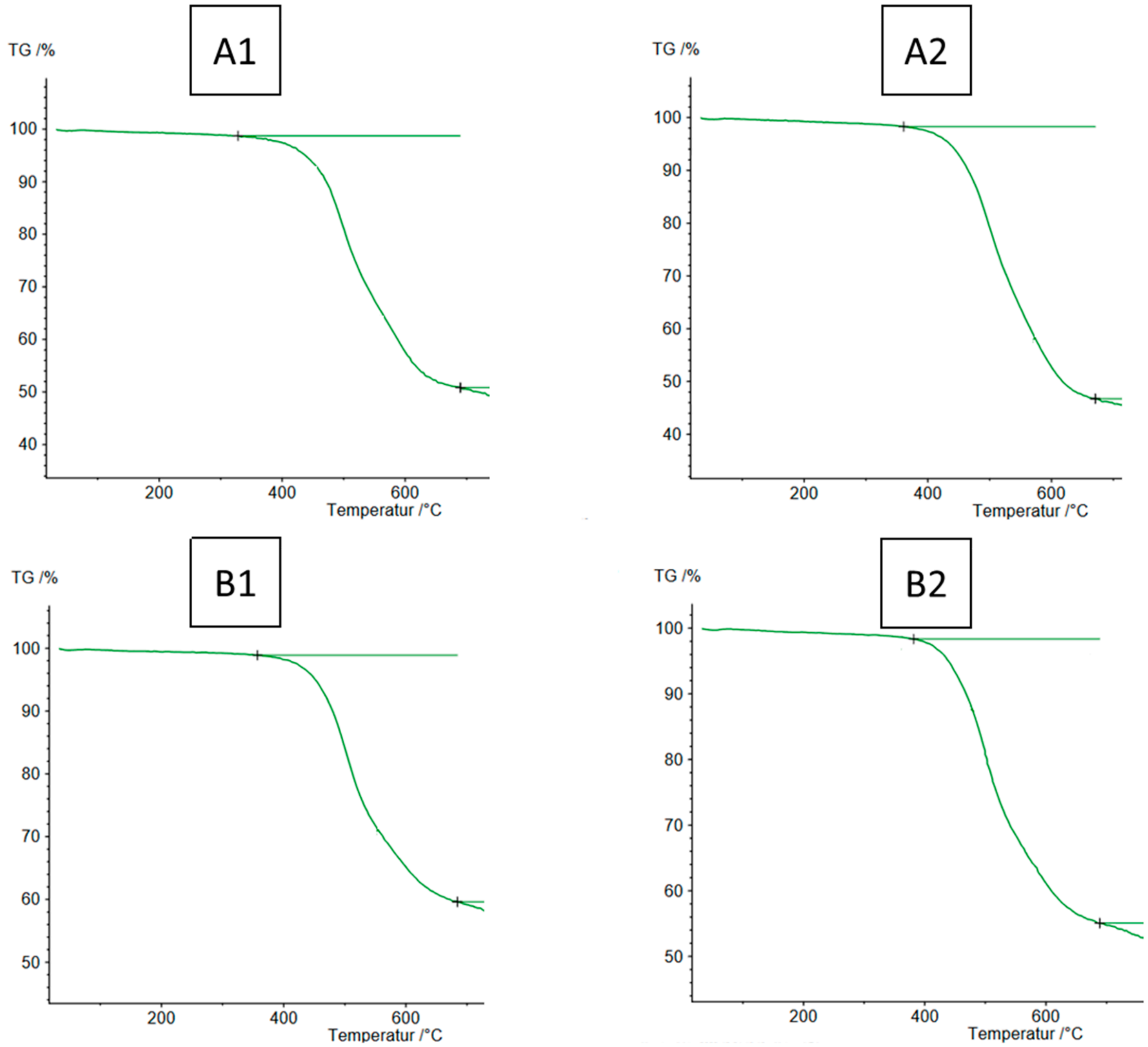

Figure S8: Highlighted are the TG results from table S3 exp. 13 (A1), exp15 (A2), exp. 3 (B1) and exp. 5 (B2).

A1 shows a decomposing temperature of 328 °C decomposing with 48% total mass loss. Lowering the density leads to 361 °C decomposing temperature with 52% total mass loss. Organic contents of the used polymer are similar with 4 % difference. On the other hand, Experiments with some MTMS content shows a higher decomposition of 357 °C with 39 % mass loss of organic content. Lowering the density leads to 382 ° decomposing temperature with 43 % mass loss. Here the difference of lost mass is about 4 %. The higher the organic content of DMDMS (one methyl group) results in higher mass loss in comparison to MTMS.

Additionally, IR spectroscopy measurements were performed as well (Figure S9) to reveal functional features and analyse of the IR active bands of the aerogels. Results are similar to PVMDMS FTIR spectra in Figure S6 and FTIR-spectra in Figure S5. In comparison between DMDMS and MTMS band at 1167 cm^{-1} in PD_4V_4VMDMS spectra disappeared which is a possible methoxy vibration, resulting in formation of a network containing aerogel.

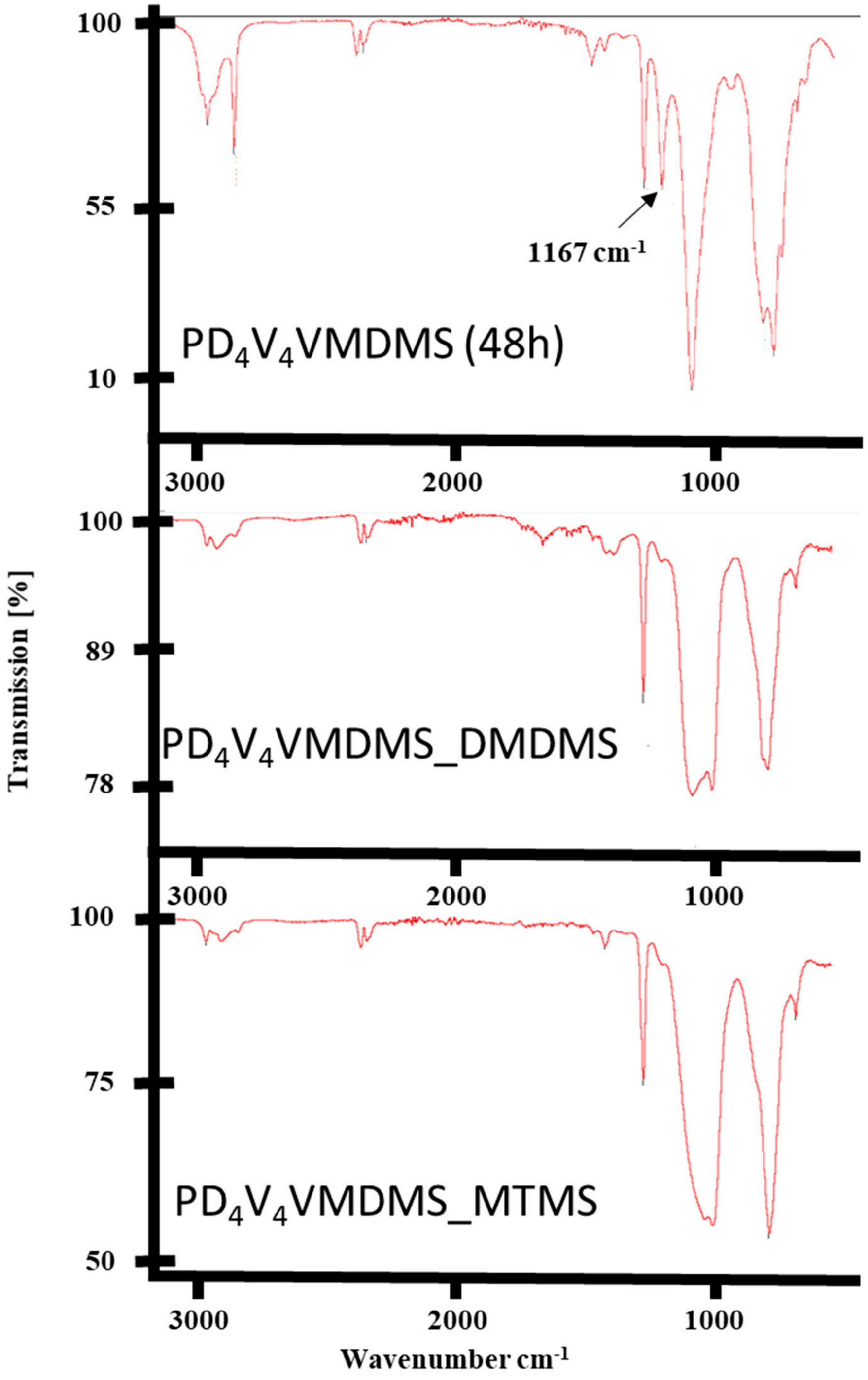

Figure S9: FTIR spectra of the prepolymer PD_4V_4VMDMS (above) and the spectra of PD_4V_4VMDMS with DMDMS (middle) or MTMS (down).

SI: Freeze Drying of D_4V_4 monolith

Originally, we were aiming for silica aerogel freeze drying evaluation and their behaviour by comparing flexible vs non-flexible aerogels of the same nature. In our freeze-drying method we used tert-butanol or cyclohexane that is sublimated from the frozen solvogel (also called cryogel) by vacuum. Hereby, phase change from solid to gas does not lead to capillary forces as well and the aerogel should be unharmed after drying [36]. Preliminary results of a D_4V_4 based monolith freeze-dried from tert-butanol are shown in Figure S10:

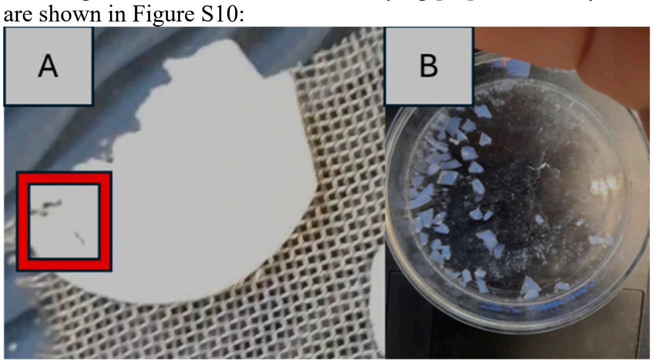

Figure S10: (A) Freeze dried D_4V_4 Sample 7. Cracks are marked with red bars. (B) Freeze dried D_4V_4 sample 2.

The flexible D_4V_4 based aerogel were obtained with a few cracks (Figure S10 (A), red bars) after freeze drying with thermal conductivities of 21 mW $m^{-1}K^{-1}$ (sample 7) and 22.4 mW $m^{-1}K^{-1}$ (sample 17). The obtained aerogels were very fragile to the point that is was difficult to measure thermal conductivity. Therefore, these samples were measured as powders. The aerogels with high density were investigated as well and show thermal conductivities of 28 mW $m^{-1}K^{-1}$ (sample 12) and 25 mW $m^{-1}K^{-1}$ (sample 2). All higher density aerogels were cracked/fragmented after the freeze drying and broken in a lot of small pieces. Testing small pieces after freezing them shows after vacuum a few cracks on the side.

Preview on freeze/dried materials

Performing a freeze drying in *t*-BuOH at other flexible organosilica aerogels is not impossible and the details will be in our next publication. There, we compare another, supercritical dried flexible aerogel from *Kanamori et. al.* from their recent publication (50 % compression) [54] and compare it with our advanced freeze-drying method. Preliminary results reveal, the freeze-dried aerogel obtained a thermal conductivity of 19.9 mW $m^{-1}K^{-1}$ (vs. supercritical drying 18 mW $m^{-1}K^{-1}$) and low density 145 g cm^{-3} (vs. supercritical drying 165 g cm^{-3}). The material shrunk 7 % after the freeze drying (Figure S11). There were no cracks and the aerogels were not brittle as the freeze dried D_4V_4 aerogels in this publication.

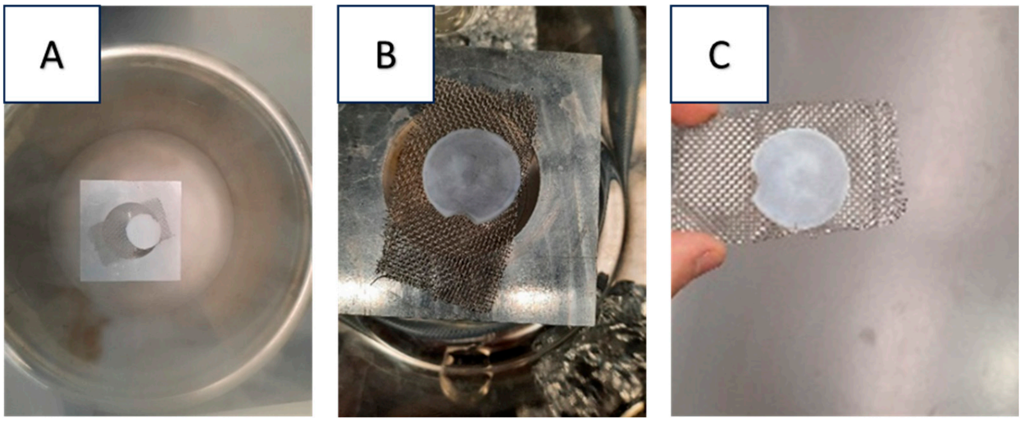

Figure S11: (A) Freezing the solvogel. (B), sublimation of tert-butanol in vacuum and (C) obtained silica-aerogel with maximal 30 % compressibility and 28 % transmittance at 550 nm.

SI: Polycondensation of PVMDMS

We used tri-clamp device with sight glas for the polycondensation reaction. Obviously a part of solvent is evaporated in time an condensed on the glass (Figure S12).

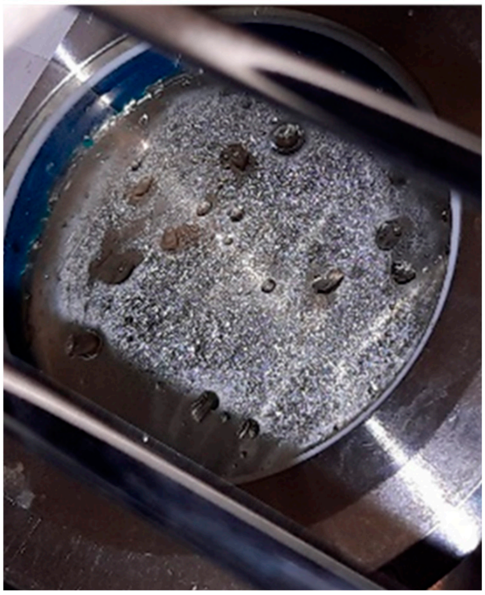

Figure S12: Used polycondensation vessel as reactor unit, with condensation media on sight glass.

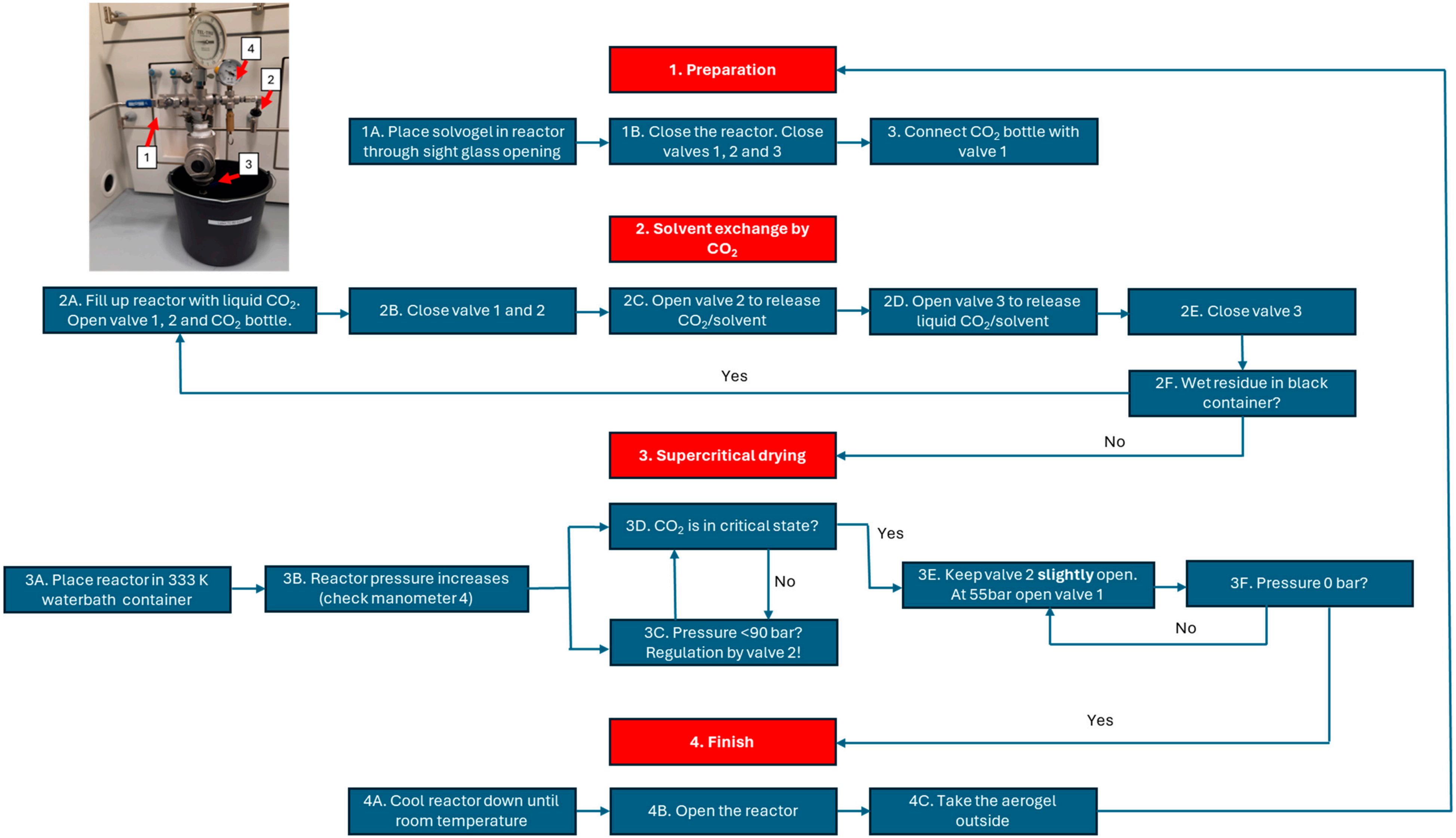

Figure S13: Scheme of the supercritical drying procedure and the used self-made critical reactor for drying step with CO_2 entry valve (1), pressure release valve (2), solvent drain valve (3) and pressure regulation unit (4). Figures of the supercritical drying steps are provided in Figure S14.

The supercritical drying is performed in suitable pressure tight reactor (Figure S13 and S14 in 1C). The Reactor is pressure tight until 120 bar and secured by a safety pressure regulation unit (4). Three openings refering to the gas entry (1) from external mobile bottle containing liquid CO_2. Second is the pressure release unit (2) and also to introduce condensation of gaseous CO_2 due to the Joule Thomson Effect. Third opening is the drain valve to remove the MeOH solvent (3).

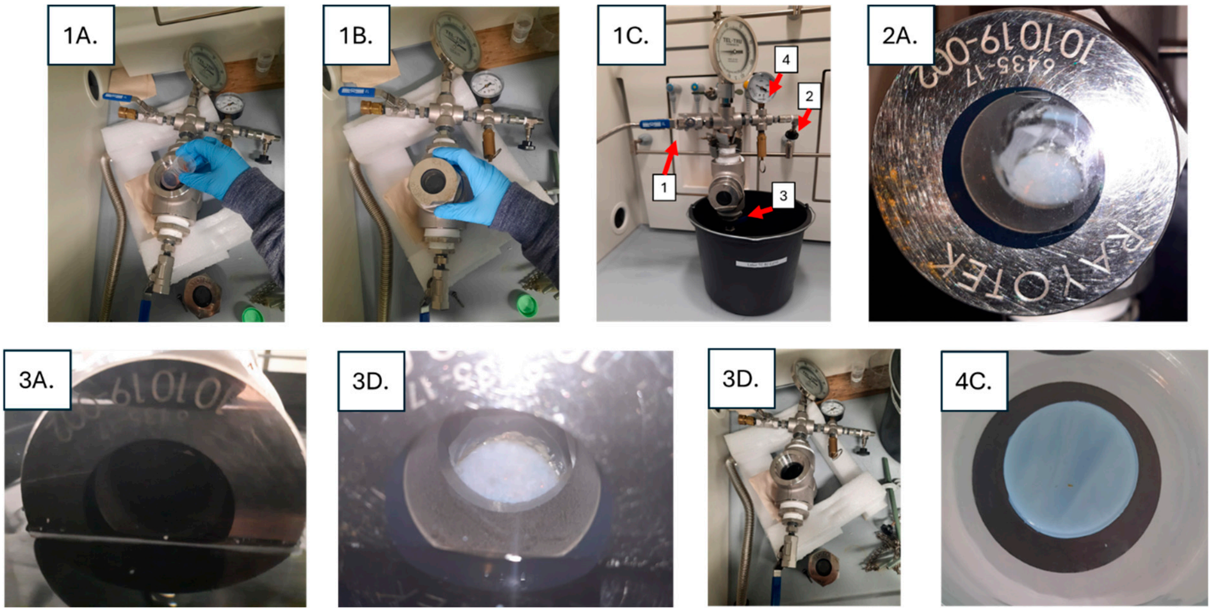

Figure S14: Pictures of the important steps during the Supercritical Drying, with respect to Figure S13.

Detailed explanation for supercritical drying: A solvogel is placed in the supercritical reactor (Figure S14, 1A) and tightly sealed by the help of teflon sealant on the cap (Sealant must be exchanged every drying step). Entry 1 valve is opened after its with the liquid CO_2 bottle connected. Valve two (2) was partly opened until liquid CO_2 was pressed from bottle to reactor, visible in the sight glass (2A). After the sight glass is completely filled with liquid CO_2 the samples were kept there for 12 hours. Afterwards the liquid CO_2 was released with valve 3 until the samples were just barely float under liquid phase. Under valve 3 a beaker can be placed there to monitor the amount of solvent (2F.), which is still left during each solvent exchange. Finally, the critical drying was done by placing a bucket with 60 °C hot water around the reactor (3A.). The gas phase shifts to supercrtical phase and pressure increases. The reactor is secured by pressure regulation unit with manometer (4). The pressure was released at 90 bar by valve 2 (Figure S14, 3B-3D) as well. Until the pressure reaches 55 bar, the valve 1 can be opened to release the pressure in CO_2 pipe connected with the closed bottle (3E). The reactor can be reopened at 0 bar and room temperature are reached (4B).

Literature

[28] Zu, G.; Shimizu, T.; Kanamori, K.; Zhu, Y.; Maeno, A.; Kaji, H. Transparent, Superflexible Doubly Cross-Linked Polyvinylpolymethylsiloxane Aerogel Superinsulators via Ambient Pressure Drying**.** *ACS. Nano.* **2018**, *12*, 1, 521–532.

[51] Liddel, U.; Becker, D. E. Infra-red spectroscopic studies of hydrogen bonding in methanol, ethanol, and t-butanol. *Spectrochimica Acta.* **1957**, *10*, 1, 70–84.

[52] Sebbar, N.; Bozzelli, J. W.; Bockhorn, H.; Kinetic Study of Di-Tert-Butyl Peroxide: Thermal Decomposition and Product Reaction Pathways, *Int. J. of Chemical Kinetics,* **2015**, *47*, *3*, 133–161.

[53] Lorand, J. P.; Radicals and scavengers. V. Steric hindrance and cage effects in the decompositions of several tert-butyl peresters. *J. Am. Chem. Soc.* **1974**, *96*, 9, 2867–2874.

[36] Zhai, S.; Yu, K.; Meng, C.; Wang, H.; Fu, J. Eco-friendly approach for preparation of hybrid silica aerogel via freeze drying method. *J. Mater. Sci.* **2022**, *57*, 7491-7502.

[54] Ueoka, R.; Hara, Y.; Maeno, A.; Kaji, H.; Nakanishi, K.; Kanamori, K. Unusual flexibility of transparent poly(methylsilsesquioxane) aerogels by surfactant-induced mesoscopic fiber-like assembly. *nat. comm.* **2024**, *461*, 1-11.
